# European lipodystrophy registry: background and structure

**DOI:** 10.1186/s13023-020-1295-y

**Published:** 2020-01-15

**Authors:** Julia von Schnurbein, Claire Adams, Baris Akinci, Giovanni Ceccarini, Maria Rosaria D’Apice, Alessandra Gambineri, Raoul C. M. Hennekam, Isabelle Jeru, Giovanna Lattanzi, Konstanze Miehle, Gabriele Nagel, Giuseppe Novelli, Ferruccio Santini, Ermelinda Santos Silva, David B. Savage, Paolo Sbraccia, Jannik Schaaf, Ekaterina Sorkina, George Tanteles, Marie-Christine Vantyghem, Camille Vatier, Corinne Vigouroux, Elena Vorona, David Araújo-Vilar, Martin Wabitsch

**Affiliations:** 1 Division of Paediatric Endocrinology and Diabetes, Department of Paediatrics and Adolescent Medicine, Centre for Rare Endocrine Disorders, Ulm University Medical Centre, Eythstraße 24, 89075 Ulm, Germany; 2 University of Cambridge Metabolic Research Laboratories, Cambridge, UK; 3 Dokuz Eylul University School of Medicine, Izmir, Turkey; 4 Obesity and Lipodystrophy Center, Endocrine Unit, University Hospital of Pisa, Pisa, Italy; 5 Medical Genetics Laboratory, Policlinico Tor Vergata, Rome, Italy; 6 Endocrinology Unit, Department of Clinical and Medical Science, S. Orsola-Malpighi Hospital, University of Bologna, Bologna, Italy; 7 Department of Paediatrics, Amsterdam University Medical Centre, Amsterdam, Netherlands; 8 Inserm U938, AP-HP, National Reference Center for Rare Diseases of Insulin Secretion and Insulin Sensitivity (PRISIS), Departments of Endocrinology, Diabetology and Reproductive Endocrinology, and Molecular Biology and Genetics, Sorbonne University, Saint-Antoine University Hospital, Paris, France; 9 CNR Institute of Molecular Genetics “Luigi Luca Cavalli-Sforza”, Unit of Bologna, Bologna, Italy; 10 Medical Department III – Endocrinology, Nephrology, Rheumatology, University of Leipzig, Leipzig, Germany; 11 Institute of Epidemiology and Medical Biometry, Ulm University, Ulm, Germany; 12 Department of Biomedicine and Prevention, University of Rome Tor Vergata - Policlinico Tor Vergata, Rome, Italy; 13 Neuromed IRCCS Institute, Pozzilli, IS Italy; 14 Pediatric Gastroenterology Unit, Pediatrics Division, Centro Materno Infantil do Norte (CMIN), Centro Hospitalar Universitário do Porto, Porto, Portugal; 15 Instituto de Ciências Biomédicas Abel Salazar (ICBAS), Universidade do Porto, Porto, Portugal; 16 UCIBIO, REQUIMTE, Laboratory of Biochemistry, Faculdade de Farmácia do Porto, Porto, Portugal; 17 Internal Medicine Unit and Obesity Center, Department of Systems Medicine, University of Rome Tor Vergata, Rome, Italy; 18 Medical Informatics Group, University Hospital Frankfurt, Frankfurt, Germany; 19 Endocrinology Research Centre, Moscow, Russia; 20 Clinical Genetics Clinic, Cyprus Institute of Neurology & Genetics, 1683 Nicosia, Republic of Cyprus; 21 CHU Lille, Department of Endocrinology, Diabetology and Metabolism, Inserm, Translational Research for Diabetes, UMR-1190, European Genomic Institute for Diabetes, University of Lille, 59000 Lille, France; 22 Division of Endocrinology, Diabetology and Nutritional Medicine, Department of Medicine B of Gastroenterology and Hepatology, University Clinics of Münster, Münster, Germany; 23 Thyroid and Metabolic Diseases Unit, Centro de Investigación en Medicina Molecular y Enfermedades Crónicas (CIMUS)-IDIS, School of Medicine, Universidade de Santiago de Compostela, Avda. Barcelona 3, 15707 Santiago de Compostela, Spain

**Keywords:** Lipodystrophy, Registry, Rare diseases, Adipose tissue

## Abstract

**Background:**

Lipodystrophy syndromes comprise a group of extremely rare and heterogeneous diseases characterized by a selective loss of adipose tissue in the absence of nutritional deprivation or catabolic state. Because of the rarity of each lipodystrophy subform, research in this area is difficult and international co-operation mandatory. Therefore, in 2016, the European Consortium of Lipodystrophies (ECLip) decided to create a registry for patients with lipodystrophy.

**Results:**

The registry was build using the information technology Open Source Registry System for Rare Diseases in the EU (OSSE), an open-source software and toolbox. Lipodystrophy specific data forms were developed based on current knowledge of typical signs and symptoms of lipodystrophy. The platform complies with the new General Data Protection Regulation (EU) 2016/679 by ensuring patient pseudonymization, informational separation of powers, secure data storage and security of communication, user authentication, person specific access to data, and recording of access granted to any data. Inclusion criteria are all patients with any form of lipodystrophy (with the exception of HIV-associated lipodystrophy). So far 246 patients from nine centres (Amsterdam, Bologna, Izmir, Leipzig, Münster, Moscow, Pisa, Santiago de Compostela, Ulm) have been recruited. With the help from the six centres on the brink of recruitment (Cambridge, Lille, Nicosia, Paris, Porto, Rome) this number is expected to double within the next one or 2 years.

**Conclusions:**

A European registry for all patients with lipodystrophy will provide a platform for improved research in the area of lipodystrophy. All physicians from Europe and neighbouring countries caring for patients with lipodystrophy are invited to participate in the ECLip Registry.

**Study registration:**

ClinicalTrials.gov (NCT03553420). Registered 14 March 2018, retrospectively registered.

## Introduction

Lipodystrophy syndromes form a heterogeneous group of diseases characterized by a selective loss of adipose tissue, sometimes associated with fat accumulation in other regions of the body, in the absence of nutritional deprivation or catabolic state [1]. Lipodystrophy syndromes have been traditionally divided into four major categories [2]: congenital generalized lipodystrophy (CGL), familial partial lipodystrophy (FPLD), acquired generalized lipodystrophy (AGL) and acquired partial lipodystrophy (APL). Additionally, systemic forms associated with progeroid or other syndromes and acquired localized forms have been described. In total, this currently subsumes about 50 different subtypes of lipodystrophy (Table 1 [3]); a number which is growing regularly, illustrating the fact that lipodystrophy can result from many different pathophysiological mechanisms targeting adipose tissue, many of which are still to be discovered.

**Table 1.** List of lipodystrophy subformes (adapted from [3]).

1 Congenital (Familial)	
1.1 Generalized (CGL)	
1.1.1 Type 1 CGL (AGPAT2, recessive, OMIM #608594)	
1.1.2 Type 2 CGL (BSCL2, recessive, OMIM #269700)	
1.1.3 Type 3 CGL (CAV1, recessive, OMIM #612526)	
1.1.4 Type 4 CGL (PTRF, recessive, OMIM #613327)	
1.1.5 PPARG -associated CGL (PPARG, recessive)	
1.1.6 CGL with progressive encephalopathy (OMIM: #615924)	
1.1.7 CGL undefined	
1.2 Partial	
1.2.1 Type 1 FPLD (Köbberling syndrome; genes unknown, OMIM %608600)	
1.2.2 Type 2 FPLD (Dunnigan disease; LMNA, (co-)dominant, OMIM #151660)	
1.2.3 Type 3 FPLD (PPARG , dominant, OMIM #604367)	
1.2.4 Type 4 FPLD (PLIN1, dominant, OMIM #613877)	
1.2.5 Type 5 FPLD (CIDEC, recessive, OMIM #615238)	
1.2.6 Type 6 FPLD (LIPE, recessive, OMIM #615980)	
1.2.7 Type 7 FPLD with congenital cataracts, and neurodegeneration (CAV1, dominant, OMIM	
1.2.8 AKT2-linked lipodystrophy (AKT2, dominant)	
1.2.9 MFN2 associated FPLD (MFN2)	
1.3 Systemic	
1.3.1 Progeroid syndromes	
1.3.1.1 Hutchinson-Gilford progeria syndrome (LMNA, dominant, OMIM #176670)	
1.3.1.2 Atypical Werner syndrome and atypical progeroid syndrome (LMNA-associated)	
1.3.1.3 SHORT syndrome (PIK3R1, dominant, OMIM #269880)	
1.3.1.4 MDPL syndrome (generalized or partial; POLD1, dominant, OMIM #615381)	
1.3.1.5 Keppen-Lubinsky syndrome (KCNJ6, dominant, OMIM #614098)	
1.3.1.6 Néstor-Guillermo progeria syndrome (BANF1, recessive, OMIM #614008)	
1.3.1.7 Mandibuloacral dysplasia, type B (ZMPSTE24, recessive, OMIM #608612)	
1.3.1.8 Ruijs-Aalfs syndrome (SPRTN, recessive, OMIM #616200)	
1.3.1.9 Cockayne syndrome type B (ERCC6, recessive, OMIM #133540)	
1.3.1.10 Cockayne syndrome type A (ERCC8, recessive, OMIM #216400)	
1.3.1.11 Lipodystrophy-intellectual disability-deafness syndrome (Rajab-Spranger syndrome, OMIM %608154)	
1.3.1.12 Marfan syndrome with neonatal progeroid –like lipodystrophy (FBN1, dominant, OMIM #616914)	
1.3.1.13 CAV1-associated neonatal onset lipodystrophy syndrome (CAV1, dominant)	
1.3.1.14 Werner syndrome (WRN/RECQL2, recessive, OMIM #277700)	
1.3.1.15 Mandibuloacral dysplasia type A (LMNA, recessive, OMIM #248370)	
1.3.1.16 PCYT1A lipodystrophy (PCYT1A, recessive)	
1.3.1.17 Wiedemann Rautenstrauch syndrome (POLR3A, recessive, OMIM #264090)	
1.3.1.18 Fontaine progeroid syndrome (SLC25A24, de novo, OMIM # 612289)	
1.3.2 Autoinflammatory syndromes, ALDD (generalized or partial)	
1.3.2.1 PRAAS 1 (PSMB8, recessive or digenic with PSMA3 or PSMB4, OMIM #256040)	
1.3.2.2 PRAAS 2 (POMP, dominant, OMIM #618048)	
1.3.2.3 PRAAS3 (PSMB4, recessive or digenic with PSMB9, OMIM #617591)	
1.3.2.4 Panniculitis-associated lipodystrophy (OTULIN, recessive, OMIM #617099)	
1.3.3 Others	
1.3.3.1. Optic atrophy, cataracts, lipodystrophy/lipoatrophy, peripheral neuropathy (OPA3, dominant, OMIM #165300)	
2 Acquired	
2.1 Generalized	
2.1.1 Acquired generalized lipodystrophy, idiopathic	
2.1.2 Acquired generalized lipodystrophy, autoimmune	
2.1.3 Acquired generalized lipodystrophy, panniculitis	
2.2 Partial (excluding HIV associated lipodystrophy)	
2.2.1 Acquired partial lipodystrophy (Barraquer-Simons syndrome, OMIM #608709)	
2.2.2 Lipodystrophy associated with total body irradiation and hematopoietic stem cell transplant	
2.2.3 Acquired partial lipodystrophy, undefined	
2.3 Localized	
2.3.1 Lipodystrophy caused by drug injections	
2.3.2 Lipodystrophy semicircularis	
2.3.3 Centrifugal lipodystrophy	
2.3.4 Progressive hemifacial atrophy (Parry Romberg syndrome, OMIM % 141300)	
2.3.5 Idiopathic localized lipodystrophy	
2.3.6 Panniculitis induced localized lipodystrophy	

*CGL* Congenital generalized lipodystrophy, *FPLD* Familial partial lipodystrophy, *PRAAS* Proteasome-associated auto-inflammatory syndrome

One major cause for comorbidities in lipodystrophy syndromes is the fat loss itself. Depending on the extent of fat loss (and also varying according to subtype, age, gender, and some yet unidentified factors) the deficient adipose mass results in ectopic lipid storage in various organs including liver and muscles which can cause a severe insulin resistance, hypertriglyceridemia, and non-alcoholic fatty liver disease. This in turn may lead to severe complications such as diabetes, cardiovascular disease, liver cirrhosis, ovarian dysfunction, and acute pancreatitis. In addition, each lipodystrophy subform has specific and greatly varying comorbidities which include muscular and/or neurological manifestations and anomalies of skin, skeleton, and internal organs. The phenotypic expressivity of each lipodystrophy subform and the associated co-morbidities and their prognosis is extremely variable, even within each category.

With the exception of HIV-associated lipodystrophy, lipodystrophy syndromes are extremely infrequent [1]. The prevalence figures that are being considered vary tremendously, largely due to the difficulty of gathering representative series of sufficient size. Thus, the prevalence for infrequent lipodystrophy subforms has been estimated as 1 in 10 million [4]. Based on the study of a series of databases of electronic medical records and analysis of European literature since 2000, the global prevalence for all lipodystrophy subforms was recently estimated as 1.3–4.7 per million, being 0.23 per million for generalized forms and 2.84 for partial forms [5]. For certain subtypes, even only a handful of cases have been reported [6, 7]. However, it is likely that lipodystrophy syndromes are underdiagnosed, especially partial forms of the disease [8–10].

The low prevalence of lipodystrophy syndromes combined with the huge heterogeneity in phenotype and genotype make it difficult, if not impossible, for a single centre or research group to describe new genetic and/or environmental determinants of specific forms and to study the natural history of these heterogeneous conditions, the associated complications and co-morbidities, the response to different management strategies, and prognosis including mortality rate.

Hence it is clear that an international approach is needed, in which multiple groups experienced in diagnostics and management of lipodystrophy syndromes participate.

In Bologna in 2014, a group of scientists devoted to lipodystrophy syndromes founded the European Consortium of Lipodystrophies, ECLip [11], as a tool that allows the exchange of knowledge, skills and ideas to advance research in the field of lipodystrophy, and that will enable the implementation of common research projects. Today, more than 30 groups from 20 European countries participate in this consortium, with annual meetings where advances and achievements of all facets in the field of lipodystrophy are presented and discussed.

In 2016, the consortium decided to found a European Registry of Lipodystrophies.

By creating one central, international registry combining data from centres all over Europe and neighbouring countries, not only can data be collected on all clinical and molecular aspects related to the syndromes, but also virtual information can be included about the availability of biological samples from the patients for basic researchers to elucidate new pathogenic mechanisms.

The aim of this manuscript is to present the goals pursued in the short, medium and long term by the European Registry of Lipodystrophies, its structure and the basis that govern its internal functioning. It is also an invitation to other groups to collaborate in this common effort to unravel the different basic and clinical aspects of lipodystrophy syndromes that to this day still remain undisclosed.

## Methods

### Aim of the registry

The aim of this registry is to enable physicians within but also outside of Europe to work together in the field of lipodystrophy and to accumulate sufficient data for sound research in this area.

This aim can be achieved by three different approaches within the registry:

- First, by striving to engage all active centres caring for patients with lipodystrophy in Europe, the registry aims to provide the basis for an improved estimate of the prevalence of lipodystrophy in Europe.
- Second, the registry offers a firm basis for patient-centred clinical lipodystrophy research, for instance on the natural course of various lipodystrophy syndromes, comorbidities, family history, and management strategies and outcomes. Thus, the registry will contribute to research areas currently not well examined due to the extreme rarity of some lipodystrophy subforms.
- Third, the registry provides a platform for nested investigations on specific clinical, molecular and functional topics. Patients with a particular lipodystrophy subform can be identified via the registry and invited for specific research projects through the physician who enrolled her or him in the registry. Due to the flexibility of the employed IT structure (see below), the results of such research projects can also be documented in the registry. This not only enables researchers of this specific project to use all previously documented information of the patients but also makes the raw data of this project available to future projects and researchers.

### Quality aspects of the registry

We attempt to reach a high level of compliance to international quality standards for rare disease registries. We adhere as closely as possible to the standard described for the two domains research quality and evidence quality as published earlier [12]. The operational aspects of the registry are based on the standards defined by the European Platform for Rare Disease Registries 2011–2014 (EPIRARE) survey [13]. In addition, the registry follows the FAIR principles [14] ensuring that (meta) data are findable, accessible, interoperable and reusable for example by using international accepted coding for diagnosis (ICD 10, OrphanCode and OMIM code) and treatment (Anatomical Therapeutic Chemical (ATC) Classification System).

To avoid duplicate entries of a patient, the data set entered for each new patient is checked automatically by the pseudonymization tool for correspondence with existing data sets (record linkage). Depending on the degree of IDAT correspondence, a new data set is created or an existing one re-turned. In addition, the Institute of Epidemiology and Medical Biometry of the Ulm University will biannually perform data plausibility checks. Retrospective data (for example from deceased patients) will be marked as such to differentiate from prospectively collected data.

### Government of the ECLip registry

The European Registry of Lipodystrophies [15] has been developed and is supported by public institutions (universities and research centres), independent of private companies. The head of the registry are the ECLip Registry members. Any physician or scientist seeing patients with lipodystrophy who wants to participate can apply at the registry board (see below) to become a member of the registry. Apart from a positive vote by the board, the centre also has to obtain approval by their local ethics committee to participate in the registry. All registry members can attend the registry members meeting, which is being hold annually as part of the ECLip meetings. There is one vote for every institution with one (or more) registry member.

All ECLip members are entitled to decide over research projects from third parties and major changes to the registry. They also elect 7–10 (currently 9) ECLip members to form the registry board for a period of 3 years.

The registry board is the governing body of the registry and responsible for managing the registry. It controls the data storage and the Local Operating Group (see below), votes on research proposals from ECLip members, improves the registry, decides who can become registry member, and processes requests to inform individual physicians about specific research results important for their patients. In addition, for each research project a contact person from the registry board will be selected who will support this specific project and will be part of the research team of this project. Furthermore, in order to guarantee the quality of the data, on a 6 months rotating basis a member of the registry board acts as curator. The curator ensures that all the data entered in the registry are correct, and that there are no crude inconsistencies between the proposed diagnosis and the phenotype described.

The registry board is being supported by a Local Operating Group consisting of a member of the Open Source Registry System for Rare Diseases in the EU (OSSE) team (see below) and the people from the Institute of Epidemiology and Medical Biometry of the Ulm University working in the project including the IT administrator. Duties of the Local Operating Group are implementation of improvements in the registry, merging and deletion of patients if necessary, creation of new users, maintenance of the data set, and support and provision of data for research projects.

As the servers for the registry are based at the University of Ulm, the stewardship of the registry data falls to the University of Ulm.

### Set-up of the ECLip registry

#### Open source registry system for rare diseases in the EU

The ECLip Registry has been set up using OSSE [16], an open source software and toolbox. The Open Source Software OSSE has been specifically designed as a framework and an organizational process to set up a rare disease specific registry [14]. The fundamental goal of OSSE is to provide patient associations, physicians and other stakeholders the opportunity to create and establish a patient registry without extensive IT knowledge.

#### Data processing components

**Fig. 1:**
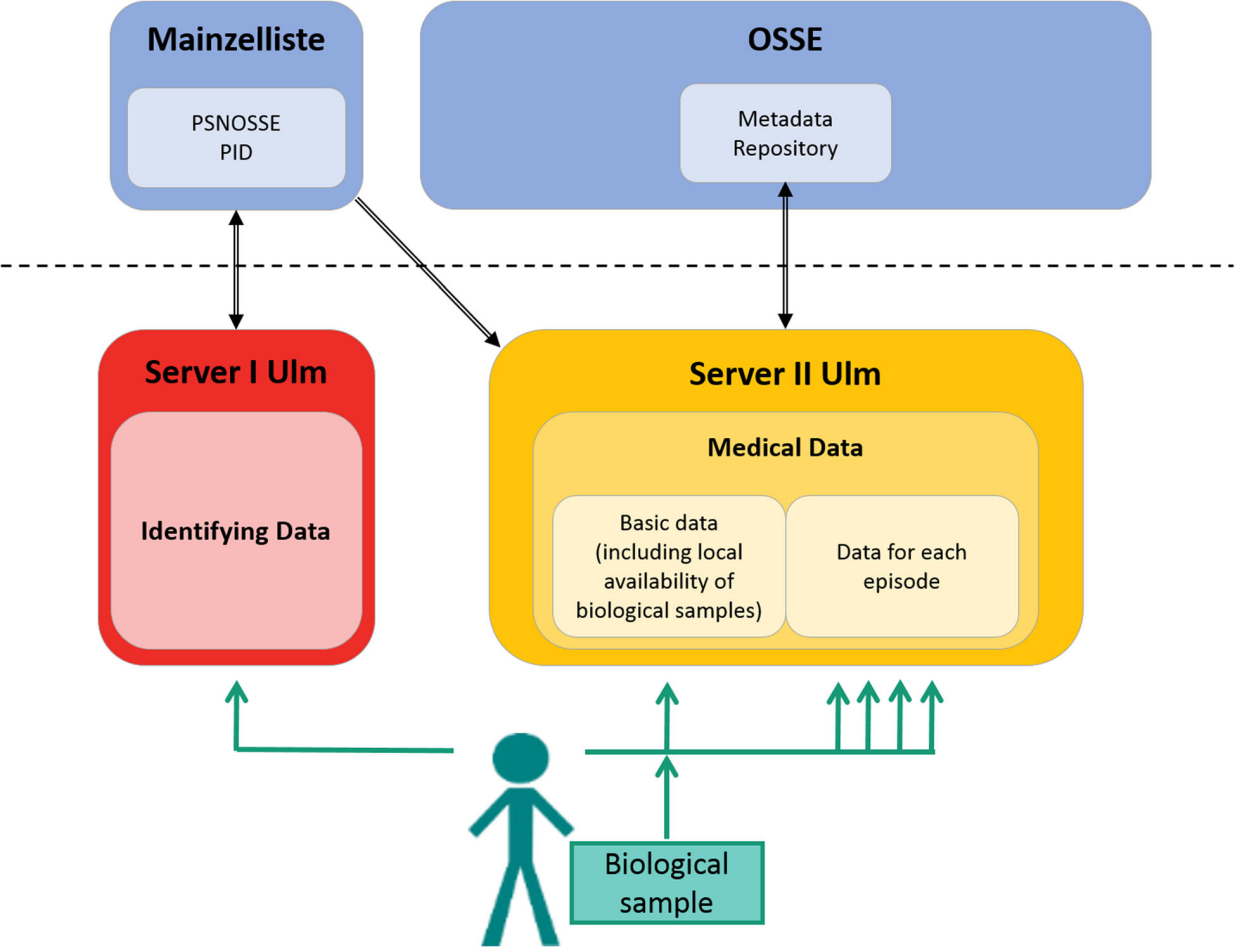
Data transfer within the ECLip Registry. Figure 1 shows data transfer within the ECLip Registry from the patient to two separate servers localized at Ulm University. The online tool Mainzelliste provides patients pseudonyms whereas the metadata repository is stored at the provider of the IT structure, OSSE. OSSE: Open Source Registry System for Rare Diseases in the EU, PID: unique patient identifier, PNSOSSE: second-level pseudonym

The OSSE registry software for the ECLip Registry provides the recording and storage of basic and longitudinal medical data of patients affected by lipodystrophy (Fig. 1). Data fields and forms are specifically designed to fit the description of lipodystrophy syndromes (Table 2). These forms and fields can be modified and supplemented throughout the running registry. Data fields used in the registry are created via the Meta Data Repository (MDR) as data elements (Fig. 1). The MDR offers a controlled vocabulary and can provide machine-readable, structured information on data elements, e.g. conceptual domains or value ranges. Data elements are used in forms that are used to enter patient data in the registry.

**Table 2:** Overview of assessments in the ECLip Registry

Identifying data	
name	
date of birth	
Basic medical data	
gender	
demographic data	
diagnosis	
genetic information if available	
death (if applicable)	
informed consent	
family history	
local availability of biological samples	
hypothesis related questions	
Data for each episode	
anthropometric data and vital signs	
adipose tissue	
skinfold values	
anatomical distribution of the lipoatrophic and lipohypertrophic regions	
analysis of body composition by dual-energy X-ray absorptiometry	
signs and symptoms in the following organ systems	
skin & appendages	
skull & face	
thyroid	
eyes & vision	
nose	
ears &hearing	
mouth	
heart & vascular system	
abdomen & gastrointestinal organs	
kidney & renal function	
neuromuscular functions	
bones & joints	
reproductive health & pregnancy	
haematology & immune system	
psychosocial health	
metabolic system and complications of diabetes	
most recent clinical laboratory values	
medication	

#### Data protection

The platform is designed to comply with all current data protection requirements specifically the General Data Protection Regulation (EU) 2016/679 by the following procedures:

### Pseudonymization

The most important procedure for data protection is patient pseudonymization. The personal identification data of the patients (IDAT) are assigned a random code (pseudonym) by using a pseudonymization tool called Mainzelliste. Mainzelliste is an open-source software to create and manage patient-pseudonyms in research projects [17]. It creates a unique identifier (PID) and a second-level pseudonym (PSNOSSE) for each patient. When a pseudonym is requested by the OSSE registry, Mainzelliste checks the data set for correspondence with existing data sets (record linkage). Depending on the degree of IDAT correspondence, a new data set is created or an existing one returned (Fig. 1).

Communication between the identity management and the OSSE registry happens via a web browser. A temporary identifier ensures that the treating physician can see the identity (ID) management’s entry mask (the patient’s name) integrated into the user interface together with the corresponding medical data (MDAT) of his or her patient. However, the returned pseudonym of the patient, “PSNOSSE”, is not shown to the physician though it is stored together with the MDAT. Thereby it becomes impossible to correlate IDAT and PSNOSSE outside the ID management system even manually (Fig. 1).

### Informational separation of powers

IDAT and the clinical information of the patients are housed on separate (and adequately protected) servers (Fig. 1). These servers are logically, physically and organizationally independent.

The institution in charge of ID management (the Institute of Epidemiology and Medical Biometry of the Ulm University) operates its own legal responsibility and is not subject to the directives of the registry management. This ensures that individuals with access to clinical or biomaterial data in the ECLip Registry outside the treatment context are not able to correlate the data to a patient.

### Access to data

Each participating physician can only access data of the patients in his or her centre, but not those of others. Access to data is also provided to each patient interested in his or her own data. For data evaluation purposes, access to pseudonymised data of a patient cohort is granted only after approval of a data evaluation concept (see below). For this purpose, the patient data are exported with a new pseudonym ensuring that the data recipient can’t see the pseudonym of the data owner. Further methods to ensure data protection included secure data storage on encrypted hard drive partitions, security of communication via encrypted connections and firewall protection, user authentication via a username and password, and recording of access granted to any data.

#### Patient recruitment; inclusion and exclusion criteria

All patients of all ages with any form of lipodystrophy presenting at a participating centre are asked to participate in the ECLip Registry. The only exclusion criterion is HIV-associated lipodystrophy. After written informed consent is reached, pseudonymised patient data can be entered into the registry. In most centres, ethical approval also covers inclusion of data of patients deceased or lost to follow-up in an anonymous way, because lipodystrophy syndromes are so rare that any information that can be gathered can be of vital importance for future patients.

#### Data collection, documentation and evaluation and statistical considerations

##### Data collection and documentation

Data entry is done at the individual locations via the web-based user interface; additionally, data can also be collected via data import interfaces. Also in this case, data pseudonymization takes place at the time-point of data entry. Based on the current knowledge about lipodystrophy syndromes including the recently published consensus paper [1], lipodystrophy specific data forms have been developed to cover all medical information relevant to patients with lipodystrophy (Table 2 and Additional file 1).

These data collection forms exist also in paper form (see Additional file 1) and can be used directly in the physician’s office at the time of presentation of the patient. They provide a valuable tool to ensure in-depth analysis of the patient’s signs and symptoms. The data can then be transferred into the electronic systems which reflect the same data forms and structure.

The information gathered within the ECLip Registry is structured into three different blocks (Fig. 1):

A first block collects the personal data of the patients (IDAT; stored separately, see above). A second block collects basic information on informed consent and permanent medical data of the patient including demographic data, diagnosis, genetic information if available, death (if applicable), family history, and local availability of biological samples (see below).

The third block collects information of each episode. An episode is defined as each clinical evaluation made of the patient over time; the numbers of episodes which can be entered for each patient are not limited. In each episode, the clinical information gathered of the patient is sorted into anthropometric data, signs and symptoms organized according to organ-systems, information on psychosocial health, most recent clinical laboratory values, and medication (Table 2 and Additional file 1).

### Virtual biobank

The ECLip Registry does not set up a biobank database. Existing samples are stored and managed locally and organizational data attached to this will be entered manually into the registry. For specific research purposes, a temporary sample collection point can be set up. A specific additional patient approval for this purpose might be applicable according to the regulations of each local ethics committee.

### Data evaluation

If an ECLip Registry member or a third party wants to use data of a cohort of patients in the registry for scientific purposes, a formal request has to be submitted to the registry board (or to the ECLip Registry members in case of a third party). The request is analysed for scientific soundness, feasibility, ethical considerations, and adherence to the aims of the registry. If the request is granted, a data usage contract is drawn up between all researchers, the ECLip Registry board and all physicians providing patient information.

Data and samples are available free of charge but a fee to refund the costs of sample handling.

(including laboratory preperation of the sample, administration, and shipment) and in case of a third party organizational support (storage, maintenance) might apply. The cost of handling samples will depend to the characteristics of the project and the type of samples.

### Statistical considerations and sample size

The ECLip Registry is conducted as a multicentre clinical registry and functions as observational study conducted as a cohort study.

One important aim is to provide a more accurate estimation of the prevalence of lipodystrophy syndromes in Europe. Here, countries like Spain where nearly all patients are being seen at least once in the reference centre in Santiago de Compostella (currently following around 200 patients) can serve as basis, even though, by the distribution of diagnosed cases within Spain, it is obvious, that in some areas lipodystrophy still is underdiagnosed also in Spain.

Another important aim of the ECLip Registry is to characterize the phenotype and genotype of the study sample by sex, age groups and region and to describe treatment patterns. Through a follow-up of the cohort it will be possible to describe the natural course of disease and to identify prognostic factors. Comparison and pooling with other data resources will be possible. These analyses will be mostly descriptive, but for specific research questions, as for example prognostic factors, sample size calculations will be performed.

With an estimate of above 500 million inhabitants in the EU (over 700 million in the geographical Europe) and an estimated prevalence rate for lipodystrophy of 1.3–4.7 per million, roughly 650 to 2350 patients with lipodystrophy are expected to live within the EU (and 910 to 3290 patients in the geographic Europe). Currently, 17 of 28 EU countries are participating in the European Consortium of Lipodystrophies itself, and 20 of 44 European countries. We therefore aim to recruit at least 50% of the lowest number estimated for all patients within the EU (> 325 patients), preferably of all of Europe (> 450). So far 246 patients from nine centres (Amsterdam, Bologna, Izmir, Leipzig, Münster, Moscow, Pisa, Santiago de Compostela, Ulm) have been recruited. With the help from the six centres on the brink of recruitment (Cambridge, Lille, Nicosia, Paris, Porto, Rome) this number is expected to double within the next 1 or 2 years.

#### Ethical considerations

The ECLip Registry tries to take into account all important ethical considerations. Each registry site has to obtain approval from their local ethics committee before participation in the registry. Participation is entirely voluntary for the patients and written informed consent has to be obtained from every participating patient (with the exception to anonymous data collection from patients deceased or lost to follow-up as described above). Patients can withdraw their consent at any time and according to their wishes, all their data will be either anonymised or deleted (except for data already used in a published study or in a study in progress).

As shown above, data protection is a fundamental aspect of the ECLip Registry. In addition, a further important aim is long-time preservation of the data to make it available not only for current but also future researchers.

#### Future plans

The ECLip Registry is designed for a horizon of 50 years. Periodically, every 5 years, a descriptive analysis and publication of the data incorporated into the registry is planned, once agreements have been reached between the corresponding parties involved. In addition, the registry is open to specific studies and collaborations with third parties, both from the pharmaceutical industry, and from basic research groups to launch research projects that advance the knowledge of the molecular basis of lipodystrophy syndromes, response to different management strategies or start-up of clinical trials with new drugs.

Many of the main expert centres in the field of lipodystrophy care and research in Europe are already involved in the ECLip Registry. Through personal contacts and by rising public awareness of this registry we aim to expand the registry further to include as many centres in Europe and neighbouring countries as possible. The registry is already registered at Clinical Trials. In the future, registering at further data banks such as RD-Connect and Orphanet are planned to further increase awareness of the registry.

The ECLip registry is also involved in the European Registries for Rare Endocrine Conditions (EuRRECa), a new project that aims to develop a core registry and an e-reporting programme for patients with rare endocrine conditions in Europe [18]. One of the ECLip Registry board members is member of a EuRRECa Expert Working Group [19] and in the future we plan to establish a functional connection between the EuRRECa eREC system and the ECLip OSSE Registry in order to systematically update the EuRRECa disease records on European patients affected by lipodystrophy using an agreed core of minimum data set.

Setting-up of the registry was encouraged by lipodystrophy patient associations such as the “Association of Families and People Affected by Lipodystrophy” (AELip). Many more national lipodystrophy patient advocacy groups are in the process of formation and they have been invited to name one common representative as a future ECLip Registry board member.

## Discussion

The creation of registries for rare diseases is a recommendation of the European Commission, and results from the objective need to have improved information about the prevalence of each of these diseases and also of their natural history, associated complications, health burden for affected individuals, response to different management strategies, and deeper knowledge of the aetiology and pathogenesis. A registry as an organized system that collects clinical and other data to a given purpose with methods of observational studies in a standardized manner is a suitable methodological approach to address the challenges of studying rare diseases [12].

Registries for rare diseases can also be an instrument for communication between professionals and affected individuals (European Union Committee of Experts on Rare Diseases (EUCERD) core recommendations on rare disease patient registration and data collection, [20]). They can serve as a tool for clinical benchmarks with the aim of improving patient care. Registries are also a useful tool to facilitate the implementation of clinical trials and through the participation of patient associations and experts, the identification of unmet needs of affected individuals and their families. By increasing awareness of a rare disease in the population and the medical community they may also help to reduce under-diagnosis and under-treatment in the future.

As concluded recently on the basis of a survey performed by a research group of the European Reference Network on Rare Endocrine Conditions (Endo-ERN) there is a need to improve the awareness and participation in existing registries [21]. Our paper therefore follows this recommendation and informs about the recently developed European Registry of Lipodystrophies.

## Conclusion

**Fig. 2:**
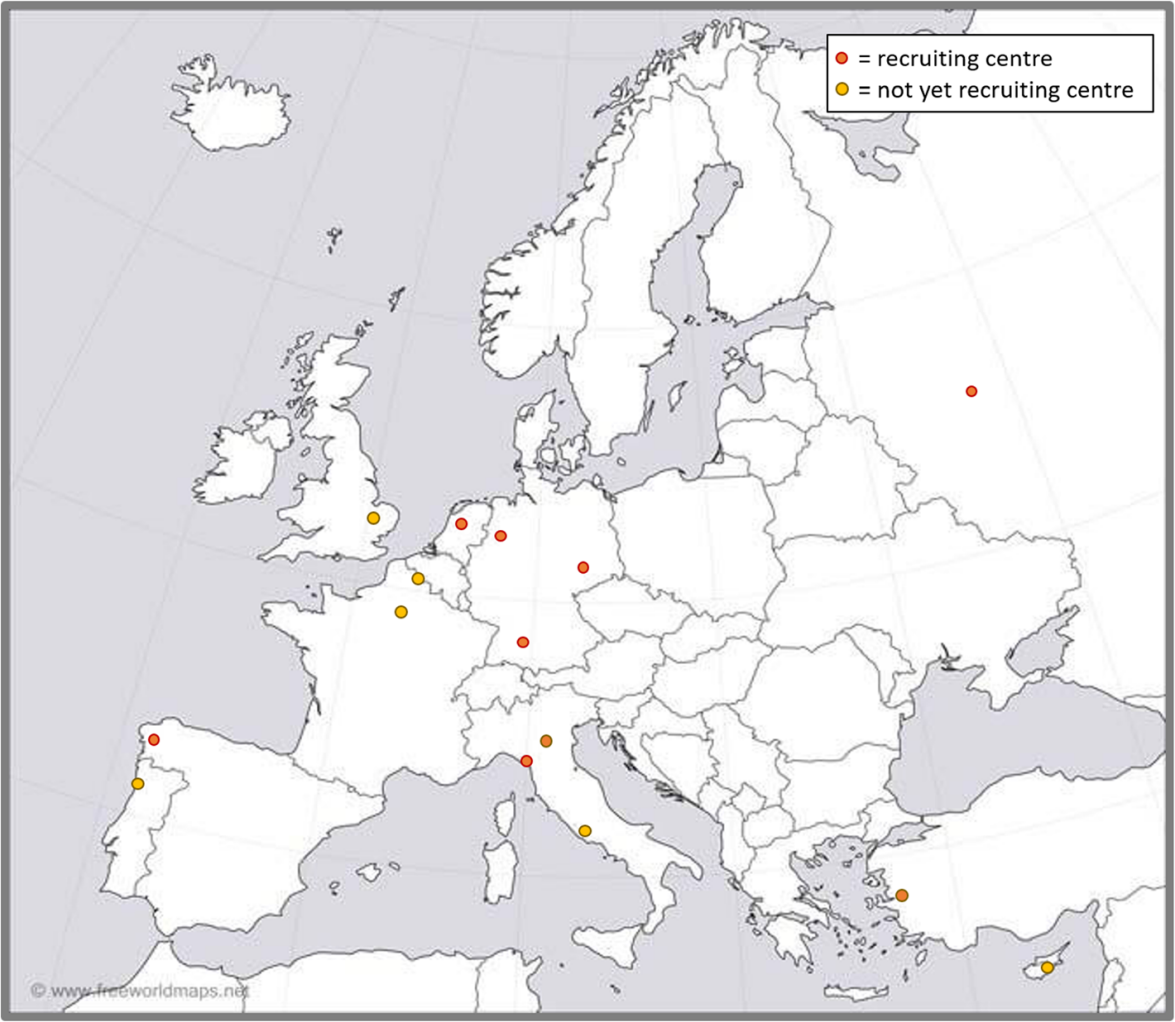
Map of all currently participating ECLip Registry centres (created from: www.freeworldmaps.net)

The ECLip Registry is today a fully operational reality [21] in which many expert centres in the field of lipodystrophy syndromes from Europe and its neighbouring countries are involved. Recruitment started on December 16th 2017; currently, 15 centres are so far participating all over Europe and neighbouring countries, with 9 centres already actively recruiting patients (Fig. 2). Six further centres are also dedicated to participate. The registry has been registered at ClinicalTrials.gov (ClinicalTrials.gov ID: NCT03553420) and is also part of EuRRECa [18].

We hope that the EClip Registry will allow in the near future to have a detailed portrait of lipodystrophy syndromes in Europe and beyond, which will result in an improvement in the welfare for and prognosis of patients affected with lipodystrophy.

To achieve this goal, we invite all physicians and researchers involved in the field of lipodystrophy to participate in this registry.

## Supplementary information

**Additional file 1.** ECLip Patient Data Sheet.

## Data Availability

All patient data are/will be stored on (two separate) servers of the Institute of Epidemiology and Medical Biometry of the Ulm University. The MDAT datasets that will be generated in this registry are not publicly available to ensure data protection. As explained above, data access is possible upon reasonable request from the ECLip Registry board. Information on what data will be collected is shown in Additional file 1 Information on OSSE, which builds the IT structure of the registry is publicly available at: www.osse-register.de/en
