# Supplementary material for: European lipodystrophy registry: background and structure
Source: Orphanet J Rare Dis. 2020 Jan 15;15:17. doi: 10.1186/s13023-020-1295-y (PMC6964101; doi:10.1186/s13023-020-1295-y)
Supplement: Supplementary file 1 — Additional file 1. ECLip Patient Data Sheet. [file 13023_2020_1295_MOESM1_ESM.docx]

**Patient form**

| **Date:** |  | **Physician:** |  |
| --- | --- | --- | --- |

**Basic**

| Name *(visible only for treating physician):* |  |
| --- | --- |
| Surname *(visible only for treating physician)*: |  |
| Date of Birth *(visible only for treating physician)*: |  |
| Gender: |  |
| Country of origin (ethnic group): |  |
| Country of birth: |  |

**Diagnosis**

| Type of Lipodystrophy: |  |
| --- | --- |
| Lipodystrophy confirmed or suspected: | ☐ confirmed (clinically or genetically) ☐ only suspected |
| ICD-10: |  |
| ORPHAN-CODE: |  |
| OMIM-CODE: |  |
| Gene *(more than one can be selected)* : |  |
| Results: | ☐ probably disease causing mutation  ☐ polymorphism  ☐ normal  ☐ not examined |
| Reference transcript identification: |  |
| Coding DNA variant (c.) combination: |  |
| Hypothetical protein change: |  |
| Reference SNP ID number: |  |
| GAffected alleles: | ☐ comp. heterozygous ☐ heterozygous ☐ homozygous |
| Familial consanguinity:  If yes, please describe | ☐ unknown ☐ no ☐ yes |
| Year patient was diagnosed: |  |
| Physician confirming diagnosis at centre: |  |
| 2^nd^ physician(s) confirming diagnosis: |  |

**Exitus**

| If yes, cause of death: |  |
| --- | --- |
| If yes, date of death: |  |

**Consent**

Options: Yes, Not answered, Denied

| ☐ | Consent for completely coded central database in Europe | ☐ | Consent for reporting incidental secondary findings |
| --- | --- | --- | --- |
| ☐ | Consent for re-contacting patients  (e.g. by E-Mail) | ☐ | Consent for storage of tissue samples |
| ☐ | Consent for local diagnostics/ research | ☐ | Consent for storage of blood samples |

**Family History**

**Lipodystrophy**

| of mother | ☐ | Unknown | ☐ | No | ☐ | Yes |  |  |
| --- | --- | --- | --- | --- | --- | --- | --- | --- |
| of father | ☐ | Unknown | ☐ | No | ☐ | Yes |  |  |
| of siblings | ☐ | n/a | ☐ | None | ☐ | Yes | If yes, number of affected siblings: |  |
| of offspring | ☐ | n/a | ☐ | None | ☐ | Yes | If yes, number of affected offspring: |  |
| of other family members | ☐ | Unknown | ☐ | None | ☐ | Yes | If yes, specify which family member: |  |
|  |  |  |  |  |  |  |  |  |

**Diabetes**

| of mother | ☐ | Unknown | ☐ | No | ☐ | Yes |  |  |
| --- | --- | --- | --- | --- | --- | --- | --- | --- |
| of father | ☐ | Unknown | ☐ | No | ☐ | Yes |  |  |
| of siblings | ☐ | n/a | ☐ | None | ☐ | Yes | If yes, number of affected siblings: |  |
| of offspring | ☐ | n/a | ☐ | None | ☐ | Yes | If yes, number of affected offspring: |  |

**Pancreatitis**

| of mother | ☐ | Unknown | ☐ | No | ☐ | Yes |  |  |
| --- | --- | --- | --- | --- | --- | --- | --- | --- |
| of father | ☐ | Unknown | ☐ | No | ☐ | Yes |  |  |
| of siblings | ☐ | n/a | ☐ | None | ☐ | Yes | If yes, number of affected siblings: |  |
| of offspring | ☐ | n/a | ☐ | None | ☐ | Yes | If yes, number of affected offspring: |  |

**NAFLD or liver cirrhosis**

| of mother | ☐ | Unknown | ☐ | No | ☐ | Yes |  |  |
| --- | --- | --- | --- | --- | --- | --- | --- | --- |
| of father | ☐ | Unknown | ☐ | No | ☐ | Yes |  |  |
| of siblings | ☐ | n/a | ☐ | None | ☐ | Yes | If yes, number of affected siblings: |  |
| of offspring | ☐ | n/a | ☐ | None | ☐ | Yes | If yes, number of affected offspring: |  |

**Cardiomyopathy**

| of mother | ☐ | Unknown | ☐ | No | ☐ | Yes |  |  |
| --- | --- | --- | --- | --- | --- | --- | --- | --- |
| of father | ☐ | Unknown | ☐ | No | ☐ | Yes |  |  |
| of siblings | ☐ | n/a | ☐ | None | ☐ | Yes | If yes, number of affected siblings: |  |
| of offspring | ☐ | n/a | ☐ | None | ☐ | Yes | If yes, number of affected offspring: |  |

**Conduction disorders**

| of mother | ☐ | Unknown | ☐ | No | ☐ | Yes |  |  |
| --- | --- | --- | --- | --- | --- | --- | --- | --- |
| of father | ☐ | Unknown | ☐ | No | ☐ | Yes |  |  |
| of siblings | ☐ | n/a | ☐ | None | ☐ | Yes | If yes, number of affected siblings: |  |
| of offspring | ☐ | n/a | ☐ | None | ☐ | Yes | If yes, number of affected offspring: |  |

**Myopathy**

| of mother | ☐ | Unknown | ☐ | No | ☐ | Yes |  |  |
| --- | --- | --- | --- | --- | --- | --- | --- | --- |
| of father | ☐ | Unknown | ☐ | No | ☐ | Yes |  |  |
| of siblings | ☐ | n/a | ☐ | None | ☐ | Yes | If yes, number of affected siblings: |  |
| of offspring | ☐ | n/a | ☐ | None | ☐ | Yes | If yes, number of affected offspring: |  |

**Other diseases**

| of mother: |  |
| --- | --- |
| of father: |  |
| of siblings: |  |
| of offspring: |  |
| of other family members: | specify disease and which family member is affected: |

**Sample availability**

☐ No samples available ☐ Unknown ☐ Samples available:

| ☐ | **DNA**  date of sample:  tissue: | ☐ | **RNA**  date of sample:  tissue: |
| --- | --- | --- | --- |
| ☐ | **Samples for proteomics**  date of sample:  tissue: | ☐ | **Fibroblasts**  date of sample:  tissue: |
| ☐ | **LCLs**  date of sample:  tissue: | ☐ | **iPSCs**  date of sample:  tissue: |
| ☐ | **Adipocytes/preadipocytes**  date of sample:  tissue: | ☐ | **Lipoma**  date of sample:  tissue: |
| ☐ | **Whole blood**  date of sample: | ☐ | **Serum**  date of sample: |

**Hypothesis related questions**

**Options:**

- Unknown

- No

- Yes, in the patient

- Yes, in the patient family

- Yes, in the patient and family

| Do you have evidence/suspicion for anticipation? |  |
| --- | --- |
| Is there an increased incidence of pain (joints, bones) in the patient/ family? |  |
| Was there early cancer in the patient/family? |  |
| Increased frequency of thyroid disease |  |
| Psychological problems with physical appearance |  |
| Initial hypoglycemia |  |
| Was the first manifestation lipoatrophy? |  |
| Was the first manifestation accumulation of fat? |  |
| Was the first manifestation muscular hypertrophy? |  |
| Was the first manifestation liver disease? |  |
| Was the first manifestation lipid alterations? |  |
| Was the first manifestation hirsutism? |  |
| Was the first manifestation infertility? |  |
| Was the first manifestation diabetes? |  |
| Others |  |

**Episode**

| **Patient:** |  | **Date of Episode:** |  | **Physician:** |  |
| --- | --- | --- | --- | --- | --- |

**Anthropometric data**

| ☐ | Not examined | |  |  |  |  |  |  |  |  |
| --- | --- | --- | --- | --- | --- | --- | --- | --- | --- | --- |
| Height: | | cm | | |  | Blood pressure right arm (diastolic): | mmHg |  | Blood pressure left arm (diastolic): | mmHg |
| Weight: | | kg | | |  | Blood pressure right arm (systolic): | mmHg |  | Waist (at the umbilical level): | mmHg |
| Heart rate: | | bpm | | |  | Waist (at the umbilical level): | cm |  | Hip (at the iliac crest level): | cm |
| Tanner stage B: | |  | | |  | Tanner stage P: |  |  | Tanner stage G |  |
| Testes volume left: | | ml | | |  | Testes volume right: | ml |  |  |  |

**Skinfolds**

| ☐ | Not examined | ☐ | Normal | | ☐ | Unchanged | | ☐ | New or changed abnormalities | |
| --- | --- | --- | --- | --- | --- | --- | --- | --- | --- | --- |
| Triceps: | | | | mm | |  | Suprailiac: | | | mm |
| (halfway between the acromion and olecranon processes) | | | | | |  | (a diagonal fold just above the iliac crest) | | | |
| Biceps: | | | | mm | |  | Thigh: | | | mm |
| (anterior midline of the upper arm over the belly of the biceps muscle) | | | | | |  | (midway between patella and inguinal fold) | | | |
| Subscapula: | | | | mm | |  | Calf: | | | mm |
| (1 to 2 cm below the inferior angle of the scapula) | | | | | |  | (a point on the medial (inside) surface of the calf, at the level of the largest circumference) | | | |

**Distribution of lipodystrophy**

| ☐ | Not examined | ☐ | Normal | ☐ | Unchanged | ☐ | New or changed abnormalities |
| --- | --- | --- | --- | --- | --- | --- | --- |

**Options:**

- Normal (N) - Lipoatrophy (La) Excess accumulation (Ea) Lipoma (Li) - Unknown (U)

| Face | age at onset: |  | Hand/Palms | age at onset: |
| --- | --- | --- | --- | --- |
| Neck | age at onset: |  | Pelvis | age at onset: |
| Thorax | age at onset: |  | Legs | age at onset: |
| Abdomen | age at onset: |  | Feet/Soles | age at onset: |
| Arms | age at onset: |  |  |  |

**Dexa Analysis**

| ☐ | Not examined | | ☐ | | Normal | | | ☐ | Unchanged | | ☐ | | New or changed results | | | |
| --- | --- | --- | --- | --- | --- | --- | --- | --- | --- | --- | --- | --- | --- | --- | --- | --- |
| Date of Dexa Analysis: | | | |  | | | |  | | | | | | | | |
| Total fat | | g | | | |  | Arms FFM | | | g | |  | | Trunk fat | g |  |
| Total body fat | | % | | | |  | Lower limbs fat | | | g | |  | | Trunk FFM | g |  |
| Total BMD | | g/cm^2^ | | | |  | Lower limbs FFM | | | g | |  | | Trunk BMD | g/cm^2^ |  |
| Total FFM | | g | | | |  | Lower limbs BMD | | | g/cm^2^ | |  | | Spine and pelvis BMD | g/cm^2^ |  |
| Fat arms | | g | | | |  | Upper arms BMD | | | g/cm^2^ | |  | |  |  |  |

**Organ systems - Skin & Appendages**

| ☐ | Not examined | ☐ | Normal | ☐ | Unchanged | ☐ | New or changed abnormalities |
| --- | --- | --- | --- | --- | --- | --- | --- |

**If abnormal:**

| Annular erythematous | ☐ | No | ☐ | Unknown | ☐ | Yes | age at onset: |
| --- | --- | --- | --- | --- | --- | --- | --- |
| Acanthosis nigricans | ☐ | No | ☐ | Unknown | ☐ | Yes | age at onset |
| Dry/stiff skin, tight/rigid skin | ☐ | No | ☐ | Unknown | ☐ | Yes | age at onset: |
| Edematous plaques | ☐ | No | ☐ | Unknown | ☐ | Yes | age at onset: |
| Erythematous nodular skin lesions | ☐ | No | ☐ | Unknown | ☐ | Yes | age at onset: |
| Flebomegaly | ☐ | No | ☐ | Unknown | ☐ | Yes | age at onset: |
| Frostbitten hands | ☐ | No | ☐ | Unknown | ☐ | Yes | age at onset: |
| Leucomelanodermic papulaes | ☐ | No | ☐ | Unknown | ☐ | Yes | age at onset: |
| Lichenified skin | ☐ | No | ☐ | Unknown | ☐ | Yes | age at onset: |
| Lipomas | ☐ | No | ☐ | Unknown | ☐ | Yes | age at onset: |
| Pachyderma | ☐ | No | ☐ | Unknown | ☐ | Yes | age at onset: |
| Panniculitis | ☐ | No | ☐ | Unknown | ☐ | Yes | age at onset: |
| Patchy hyperpigmentation | ☐ | No | ☐ | Unknown | ☐ | Yes | age at onset: |
| Scleroderma-like changes | ☐ | No | ☐ | Unknown | ☐ | Yes | age at onset: |
| Skin atrophy | ☐ | No | ☐ | Unknown | ☐ | Yes | age at onset: |
| Skin fissures | ☐ | No | ☐ | Unknown | ☐ | Yes | age at onset: |
| Soft tissue calcinosis | ☐ | No | ☐ | Unknown | ☐ | Yes | age at onset: |
| Telangiectases | ☐ | No | ☐ | Unknown | ☐ | Yes | age at onset: |
| Xanthomata | ☐ | No | ☐ | Unknown | ☐ | Yes | age at onset: |
| Loss of hair/baldness | ☐ | No | ☐ | Unknown | ☐ | Yes | age at onset: |
| Loss of eyebrows/eyelashes | ☐ | No | ☐ | Unknown | ☐ | Yes | age at onset: |
| Hirsutism | ☐ | No | ☐ | Unknown | ☐ | Yes | age at onset: |
| Hypertrichosis | ☐ | No | ☐ | Unknown | ☐ | Yes | age at onset: |
| Early greying | ☐ | No | ☐ | Unknown | ☐ | Yes | age at onset: |
| Dysplastic nails | ☐ | No | ☐ | Unknown | ☐ | Yes | age at onset: |
| Tumors | ☐ | No | ☐ | Unknown | ☐ | Yes | type of tumor  age at onset: |
| Others |  |  |  |  |  |  | age at onset: |

**Organ systems - Skull & Face**

| ☐ | Not examined | ☐ | Normal | ☐ | Unchanged | ☐ | New or changed abnormalities |
| --- | --- | --- | --- | --- | --- | --- | --- |

If abnormal:

| Delayed closure of cranial sutures | ☐ | No | ☐ | Unknown | ☐ | Yes | age at onset: |
| --- | --- | --- | --- | --- | --- | --- | --- |
| Microcephaly | ☐ | No | ☐ | Unknown | ☐ | Yes | age at onset: |
| Apparent macrocephaly | ☐ | No | ☐ | Unknown | ☐ | Yes | age at onset: |

| Acromegaloid features | ☐ | No | ☐ | Unknown | ☐ | Yes | age at onset: |
| --- | --- | --- | --- | --- | --- | --- | --- |
| Mandibular osteolysis | ☐ | No | ☐ | Unknown | ☐ | Yes | age at onset: |
| Mandibular hypoplasia | ☐ | No | ☐ | Unknown | ☐ | Yes | age at onset: |
| Large mandible | ☐ | No | ☐ | Unknown | ☐ | Yes | age at onset: |
| Prominent forehead | ☐ | No | ☐ | Unknown | ☐ | Yes | age at onset: |
| Chin dimple | ☐ | No | ☐ | Unknown | ☐ | Yes | age at onset: |
| Short philtrum | ☐ | No | ☐ | Unknown | ☐ | Yes | age at onset: |
| Triangular face | ☐ | No | ☐ | Unknown | ☐ | Yes | age at onset: |
| Elfin facies | ☐ | No | ☐ | Unknown | ☐ | Yes | age at onset: |

**Others (Skull & Face)**

| Tumors | ☐ | No | ☐ | Unknown | ☐ | Yes | type of tumor  age at onset: |
| --- | --- | --- | --- | --- | --- | --- | --- |
| Others |  |  |  |  |  |  | age at onset: |

**Organ systems - Thyroid**

| ☐ | Not examined | ☐ | Normal | ☐ | Unchanged | ☐ | New or changed abnormalities |
| --- | --- | --- | --- | --- | --- | --- | --- |

**If abnormal:**

| Goiter | ☐ | No | ☐ | Unknown | ☐ | Yes | age at onset: |
| --- | --- | --- | --- | --- | --- | --- | --- |
| Autoimmunthyroiditis | ☐ | No | ☐ | Unknown | ☐ | Yes | age at onset: |
| Tumors | ☐ | No | ☐ | Unknown | ☐ | Yes | type of tumor  age at onset: |
| Others |  |  |  |  |  |  | age at onset: |

**Organ systems - Eyes & Vision**

| ☐ | Not examined | ☐ | Normal | ☐ | Unchanged | ☐ | New or changed abnormalities |
| --- | --- | --- | --- | --- | --- | --- | --- |

**If abnormal:**

| Proptosis | ☐ | No | ☐ | Unknown | ☐ | Yes | age at onset: |
| --- | --- | --- | --- | --- | --- | --- | --- |
| Short palpebral fissures | ☐ | No | ☐ | Unknown | ☐ | Yes | age at onset: |
| Retinopathy | ☐ | No | ☐ | Unknown | ☐ | Yes | type:  severity:  age at onset: |
| Blindness | ☐ | No | ☐ | Unknown | ☐ | Yes | age at onset: |
| Cataract | ☐ | No | ☐ | Unknown | ☐ | Yes | age at onset: |
| Periorbital swelling due to violaceous, plaques on the eyelids | ☐ | No | ☐ | Unknown | ☐ | Yes | age at onset: |
| Conjunctivitis | ☐ | No | ☐ | Unknown | ☐ | Yes | age at onset: |
| Episcleritis | ☐ | No | ☐ | Unknown | ☐ | Yes | age at onset: |
| Nystagmus | ☐ | No | ☐ | Unknown | ☐ | Yes | age at onset: |
| Ocular dysmetria | ☐ | No | ☐ | Unknown | ☐ | Yes | age at onset: |
| Deep-set eyes | ☐ | No | ☐ | Unknown | ☐ | Yes | age at onset: |
| Rieger anomaly | ☐ | No | ☐ | Unknown | ☐ | Yes | age at onset: |
| Glaucoma | ☐ | No | ☐ | Unknown | ☐ | Yes | age at onset: |
| Tumors | ☐ | No | ☐ | Unknown | ☐ | Yes | type of tumor  age at onset: |
| Others |  |  |  |  |  |  | age at onset: |

**Organ systems - Nose**

| ☐ | Not examined | ☐ | Normal | ☐ | Unchanged | ☐ | New or changed abnormalities |
| --- | --- | --- | --- | --- | --- | --- | --- |

**If abnormal:**

| Convex nasal ridge | ☐ | No | ☐ | Unknown | ☐ | Yes | age at onset: |
| --- | --- | --- | --- | --- | --- | --- | --- |
| Beak nose | ☐ | No | ☐ | Unknown | ☐ | Yes | age at onset: |
| Pointed nose | ☐ | No | ☐ | Unknown | ☐ | Yes | age at onset: |
| Wide nasal bridge | ☐ | No | ☐ | Unknown | ☐ | Yes | age at onset: |
| Hypoplastic nasal alae | ☐ | No | ☐ | Unknown | ☐ | Yes | age at onset: |
| Wide nostrils | ☐ | No | ☐ | Unknown | ☐ | Yes | age at onset: |
| Tumors | ☐ | No | ☐ | Unknown | ☐ | Yes | type of tumor  age at onset: |
| Others |  |  |  |  |  |  | age at onset: |

**Organ systems - Ears & Hearing**

| ☐ | Not examined | ☐ | Normal | ☐ | Unchanged | ☐ | New or changed abnormalities |
| --- | --- | --- | --- | --- | --- | --- | --- |

**If abnormal:**

| Large ears | ☐ | No | ☐ | Unknown | ☐ | Yes | age at onset: |
| --- | --- | --- | --- | --- | --- | --- | --- |
| Low set ears | ☐ | No | ☐ | Unknown | ☐ | Yes | age at onset: |
| Dysplastic ears | ☐ | No | ☐ | Unknown | ☐ | Yes | age at onset: |
| Absent ear lobes | ☐ | No | ☐ | Unknown | ☐ | Yes | age at onset: |
| Hypoacusia | ☐ | No | ☐ | Unknown | ☐ | Yes | conductive:  sensorineural:  age at onset: |
| Deafness | ☐ | No | ☐ | Unknown | ☐ | Yes | conductive:  sensorineural:  age at onset: |
| Tumors | ☐ | No | ☐ | Unknown | ☐ | Yes | type of tumor  age at onset: |
| Others |  |  |  |  |  |  | age at onset: |

**Organ systems - Mouth**

| ☐ | Not examined | ☐ | Normal | ☐ | Unchanged | ☐ | New or changed abnormalities |
| --- | --- | --- | --- | --- | --- | --- | --- |

**If abnormal:**

| Restricted opening of mouth | ☐ | No | ☐ | Unknown | ☐ | Yes | age at onset: |
| --- | --- | --- | --- | --- | --- | --- | --- |
| Large mouth | ☐ | No | ☐ | Unknown | ☐ | Yes | age at onset: |
| Small mouth | ☐ | No | ☐ | Unknown | ☐ | Yes | age at onset: |
| Thick lips | ☐ | No | ☐ | Unknown | ☐ | Yes | age at onset: |
| High-arched palate | ☐ | No | ☐ | Unknown | ☐ | Yes | age at onset: |
| Cleft palate | ☐ | No | ☐ | Unknown | ☐ | Yes | age at onset: |
| Dental crowding | ☐ | No | ☐ | Unknown | ☐ | Yes | age at onset: |
| Loss of teeth | ☐ | No | ☐ | Unknown | ☐ | Yes | age at onset: |
| Premature eruption of teeth | ☐ | No | ☐ | Unknown | ☐ | Yes | age at onset: |
| Hypoplastic teeth | ☐ | No | ☐ | Unknown | ☐ | Yes | age at onset: |
| Macroglossia | ☐ | No | ☐ | Unknown | ☐ | Yes | age at onset: |
| Gingival hyperplasia | ☐ | No | ☐ | Unknown | ☐ | Yes | age at onset: |
| Tumors | ☐ | No | ☐ | Unknown | ☐ | Yes | type of tumor  age at onset: |
| \| Others \|  \|  \|  \|  \|  \|  \| age at onset: \| \| --- \| --- \| --- \| --- \| --- \| --- \| --- \| --- \| |  |  |  |  |  |  | age at onset: |
|  |  |  |  |  |  |  |  |

**Organ systems – Heart**

| ☐ | Not examined | ☐ | Normal | ☐ | Unchanged | ☐ | New or changed abnormalities |
| --- | --- | --- | --- | --- | --- | --- | --- |

**If abnormal:**

| Dilated cardiomyopathy | ☐ | No | ☐ | Unknown | ☐ | Yes | age at onset:  NYHA-class: |
| --- | --- | --- | --- | --- | --- | --- | --- |
| Hypertrophic cardiomyopathy | ☐ | No | ☐ | Unknown | ☐ | Yes | age at onset:  NYHA-class: |
| Ischemic cardiomyopathy | ☐ | No | ☐ | Unknown | ☐ | Yes | age at onset:  NYHA-class: |
| Conduction abnormalities | ☐ | No | ☐ | Unknown | ☐ | Yes | age at onset:  NYHA-class: |
| Valvulopathy | ☐ | No | ☐ | Unknown | ☐ | Yes | age at onset:  NYHA-class: |
| Cardiac procedures | ☐ | No | ☐ | Unknown | ☐ | Yes |  |
| Procedure | ☐ | No | ☐ | Unknown | ☐ | Yes | description*:  date: |
| Catheterization | ☐ | No | ☐ | Unknown | ☐ | Yes | description*:  date: |
| By-pass | ☐ | No | ☐ | Unknown | ☐ | Yes | description*:  date: |
| Stent | ☐ | No | ☐ | Unknown | ☐ | Yes | description*:  date: |
| Defibrillator | ☐ | No | ☐ | Unknown | ☐ | Yes | description*:  date: |
| Heart transplant | ☐ | No | ☐ | Unknown | ☐ | Yes | description*:  date: |
| Tumors | ☐ | No | ☐ | Unknown | ☐ | Yes | type of tumor  age at onset: |
| Others |  |  |  |  |  |  | age at onset: |

*Description cardiac procedures:

|  |
| --- |
|  |

**Organ systems - Vascular**

| ☐ | Not examined | ☐ | Normal | ☐ | Unchanged | ☐ | New or changed abnormalities |
| --- | --- | --- | --- | --- | --- | --- | --- |

**If abnormal:**

| High blood pressure | ☐ | No | ☐ | Unknown | ☐ | Yes | age at onset: |
| --- | --- | --- | --- | --- | --- | --- | --- |
| Pulmonary hypertension | ☐ | No | ☐ | Unknown | ☐ | Yes | age at onset |
| Peripheral arteriopathy | ☐ | No | ☐ | Unknown | ☐ | Yes | age at onset: |
| Ischemic ulcers | ☐ | No | ☐ | Unknown | ☐ | Yes | age at onset: |
| Flebomegaly | ☐ | No | ☐ | Unknown | ☐ | Yes | age at onset: |
| Orthostatic hypotension | ☐ | No | ☐ | Unknown | ☐ | Yes | age at onset: |
| Amputation | ☐ | No | ☐ | Unknown | ☐ | Yes | date:  limb: |
| Tumors | ☐ | No | ☐ | Unknown | ☐ | Yes | type of tumor  age at onset: |
| Others |  |  |  |  |  |  | age at onset: |

**Organ systems - Abdomen external, Gastrointestinal, Hepatic/Spleen, Pancreas**

**Abdomen external**

| ☐ | Not examined |  | ☐ | Normal | ☐ | Unchanged | ☐ | New or changed abnormalities |
| --- | --- | --- | --- | --- | --- | --- | --- | --- |

If abnormal:

| Umbilical hernia | ☐ | No | ☐ | Unknown | ☐ | Yes | age at onset: |
| --- | --- | --- | --- | --- | --- | --- | --- |
| Prominent umbilicus | ☐ | No | ☐ | Unknown | ☐ | Yes | age at onset: |

**Gastrointestinal**

| ☐ | Not examined |  | ☐ | Normal | ☐ | Unchanged | ☐ | New or changed abnormalities |
| --- | --- | --- | --- | --- | --- | --- | --- | --- |

If abnormal:

| Voracious appetite | ☐ | No | ☐ | Unknown | ☐ | Yes | age at onset: |
| --- | --- | --- | --- | --- | --- | --- | --- |
| Hyperphagia | ☐ | No | ☐ | Unknown | ☐ | Yes | age at onset: |
| Poor feeding | ☐ | No | ☐ | Unknown | ☐ | Yes | age at onset: |
| Dysphagia | ☐ | No | ☐ | Unknown | ☐ | Yes | age at onset: |
| Constipation | ☐ | No | ☐ | Unknown | ☐ | Yes | age at onset: |
| Ileus | ☐ | No | ☐ | Unknown | ☐ | Yes | age at onset: |
| Esophageal dilatation | ☐ | No | ☐ | Unknown | ☐ | Yes | age at onset: |
| Esophageal dysmotility | ☐ | No | ☐ | Unknown | ☐ | Yes | age at onset: |
| Hypertrophic pyloric stenosis | ☐ | No | ☐ | Unknown | ☐ | Yes | age at onset: |

**Hepatic/Spleen**

| ☐ | Not examined |  | ☐ | Normal | ☐ | Unchanged | ☐ | New or changed abnormalities |
| --- | --- | --- | --- | --- | --- | --- | --- | --- |

If abnormal:

| Hepatomegaly | ☐ | No | ☐ | Unknown | ☐ | Yes | age at onset: |
| --- | --- | --- | --- | --- | --- | --- | --- |
| Fatty liver | ☐ | No | ☐ | Unknown | ☐ | Yes | age at onset: |
| NASH | ☐ | No | ☐ | Unknown | ☐ | Yes | age at onset: |
| Cirrhosis | ☐ | No | ☐ | Unknown | ☐ | Yes | age at onset: |
| Splenomegaly | ☐ | No | ☐ | Unknown | ☐ | Yes | age at onset: |
| Liver transplant | ☐ | No | ☐ | Unknown | ☐ | Yes | date: |

**Pancreas**

| ☐ | Not examined |  | ☐ | Normal | ☐ | Unchanged | ☐ | New or changed abnormalities |
| --- | --- | --- | --- | --- | --- | --- | --- | --- |

If abnormal:

| Pancreatitis | ☐ | No | ☐ | Unknown | ☐ | Yes | age at onset:  type: |
| --- | --- | --- | --- | --- | --- | --- | --- |

**Abdomen external, Gastrointestinal, Hepatic/Spleen, Pancreas**

| Tumors | ☐ | No | ☐ | Unknown | ☐ | Yes | type of tumor  age at onset: |
| --- | --- | --- | --- | --- | --- | --- | --- |
| Others |  |  |  |  |  |  | age at onset: |

**Organ systems - Neuromuscular**

| ☐ | Not examined |  | ☐ | Normal | ☐ | Unchanged | ☐ | New or changed abnormalities |
| --- | --- | --- | --- | --- | --- | --- | --- | --- |

**If abnormal:**

| Calf hypertrophy | ☐ | No | ☐ | Unknown | ☐ | Yes | age at onset: |
| --- | --- | --- | --- | --- | --- | --- | --- |
| Carpal tunnel syndrome | ☐ | No | ☐ | Unknown | ☐ | Yes | age at onset: |
| Contractures | ☐ | No | ☐ | Unknown | ☐ | Yes | age at onset: |
| Cranial nerves palsy | ☐ | No | ☐ | Unknown | ☐ | Yes | age at onset: |
| Encephalopathy | ☐ | No | ☐ | Unknown | ☐ | Yes | age at onset: |
| Extensor plantar responses | ☐ | No | ☐ | Unknown | ☐ | Yes | age at onset: |
| Hypermusculation | ☐ | No | ☐ | Unknown | ☐ | Yes | age at onset: |
| Hyperreflexia | ☐ | No | ☐ | Unknown | ☐ | Yes | age at onset: |
| Hypertonia | ☐ | No | ☐ | Unknown | ☐ | Yes | age at onset: |
| Mental retardation | ☐ | No | ☐ | Unknown | ☐ | Yes | age at onset: |
| Muscle atrophy | ☐ | No | ☐ | Unknown | ☐ | Yes | age at onset: |
| Muscle hypertrophy | ☐ | No | ☐ | Unknown | ☐ | Yes | age at onset: |
| Muscle rippling | ☐ | No | ☐ | Unknown | ☐ | Yes | age at onset: |
| Muscle weakness | ☐ | No | ☐ | Unknown | ☐ | Yes | age at onset: |
| Muscular pain/myalgia | ☐ | No | ☐ | Unknown | ☐ | Yes | age at onset: |
| Neurodegeneration | ☐ | No | ☐ | Unknown | ☐ | Yes | age at onset: |
| Percussion-induced muscle mounding | ☐ | No | ☐ | Unknown | ☐ | Yes | age at onset: |
| Polineuropathy | ☐ | No | ☐ | Unknown | ☐ | Yes | age at onset: |
| Seizures | ☐ | No | ☐ | Unknown | ☐ | Yes | age at onset: |
| Speech delay | ☐ | No | ☐ | Unknown | ☐ | Yes | age at onset: |
| Stroke | ☐ | No | ☐ | Unknown | ☐ | Yes | age at onset: |
| Tumors | ☐ | No | ☐ | Unknown | ☐ | Yes | age at onset:  type of tumor: |
| Others |  |  |  |  |  |  | age at onset: |

**Organ systems - Bones & Joints**

**Bones & Joints**

| ☐ | Not examined |  | ☐ | Normal | ☐ | Unchanged | ☐ | New or changed abnormalities |
| --- | --- | --- | --- | --- | --- | --- | --- | --- |

If abnormal:

| Osteopenia/osteoporosis | ☐ | No | ☐ | Unknown | ☐ | Yes | age at onset:  Z-score: |
| --- | --- | --- | --- | --- | --- | --- | --- |
| Advanced bone age | ☐ | No | ☐ | Unknown | ☐ | Yes | bone age: |
| Spinal rigidity | ☐ | No | ☐ | Unknown | ☐ | Yes | age at onset: |
| Scoliosis | ☐ | No | ☐ | Unknown | ☐ | Yes | age at onset: |
| Hyperlordosis | ☐ | No | ☐ | Unknown | ☐ | Yes | age at onset: |
| Acroosteolysis | ☐ | No | ☐ | Unknown | ☐ | Yes | age at onset: |
| Atlanto-axial instability | ☐ | No | ☐ | Unknown | ☐ | Yes | age at onset: |
| Hyperextensibility of joints | ☐ | No | ☐ | Unknown | ☐ | Yes | age at onset:  location: |
| Lytic cystic lesions | ☐ | No | ☐ | Unknown | ☐ | Yes | age at onset:  location: |
| Joint contractions | ☐ | No | ☐ | Unknown | ☐ | Yes | age at onset:  location: |
| Bone resorption (phalanges, clavicle) | ☐ | No | ☐ | Unknown | ☐ | Yes | age at onset:  location:: |
| Short stature | ☐ | No | ☐ | Unknown | ☐ | Yes |  |
| Tall stature | ☐ | No | ☐ | Unknown | ☐ | Yes |  |
| Others |  |  |  |  |  |  | age at onset: |

**Hands**

| ☐ | Not examined |  | ☐ | Normal | ☐ | Unchanged | ☐ | New or changed abnormalities |
| --- | --- | --- | --- | --- | --- | --- | --- | --- |

If abnormal:

| large hands | ☐ | No | ☐ | Unknown | ☐ | Yes | age at onset: |
| --- | --- | --- | --- | --- | --- | --- | --- |
| fingertip rounding | ☐ | No | ☐ | Unknown | ☐ | Yes | age at onset: |
| finger deformities | ☐ | No | ☐ | Unknown | ☐ | Yes | age at onset: |

**Feet**

| ☐ | Not examined |  | ☐ | Normal | ☐ | Unchanged | ☐ | New or changed abnormalities |
| --- | --- | --- | --- | --- | --- | --- | --- | --- |

If abnormal:

| large feet | ☐ | No | ☐ | Unknown | ☐ | Yes | age at onset: |
| --- | --- | --- | --- | --- | --- | --- | --- |
| rocker-bottom feet | ☐ | No | ☐ | Unknown | ☐ | Yes | age at onset: |

**Bones & Joints, Hands, Feet**

| Tumors | ☐ | No | ☐ | Unknown | ☐ | Yes | type of tumor  age at onset: |
| --- | --- | --- | --- | --- | --- | --- | --- |
| Others |  |  |  |  |  |  | age at onset: |

**Organ systems - Reproductive**

| ☐ | Not examined |  | ☐ | Normal | ☐ | Unchanged | ☐ | New or changed abnormalities |
| --- | --- | --- | --- | --- | --- | --- | --- | --- |

If abnormal:

| Precocious adrenarche | ☐ | No | ☐ | Unknown | ☐ | Yes | age at onset: |
| --- | --- | --- | --- | --- | --- | --- | --- |
| Precoccious pubarche | ☐ | No | ☐ | Unknown | ☐ | Yes | age at onset: |
| Precoccious thelarche | ☐ | No | ☐ | Unknown | ☐ | Yes | age at onset: |
| Delayed puberty | ☐ | No | ☐ | Unknown | ☐ | Yes | age at onset: |
| Infertility | ☐ | No | ☐ | Unknown | ☐ | Yes | age at onset: |
| Gender | ☐ | **Male** |  |  |  |  |  |
|  | Cryptorchidism | | ☐ | Unknown | ☐ | No | ☐ Yes |
|  | Large penis | | ☐ | Unknown | ☐ | No | ☐ Yes |
|  | Hypospadias | | ☐ | Unknown | ☐ | No | ☐ Yes |
|  | ☐ | **Female** |  |  |  |  |  |
|  | Age at menarche  Age of menopause: | |  |  |  |  |  |
|  | Oligomenorrhea | | ☐ | Unknown | ☐ | No | ☐ Yes |
|  | Precoccius thelarche | | ☐ | Unknown | ☐ | No | ☐ Yes |
|  | Amenorrhea | | ☐ | Unknown | ☐ | No | ☐ Yes |
|  | PCOS | | ☐ | Unknown | ☐ | No | ☐ Yes |
|  | Labial pseudohypertrophy | | ☐ | Unknown | ☐ | No | ☐ Yes |
|  | Clitoromegaly | | ☐ | Unknown | ☐ | No | ☐ Yes |
| Tumors | ☐ | No | ☐ | Unknown | ☐ | Yes | type of tumor  age at onset: |
| Others |  |  |  |  |  |  | age at onset: |

| **Pregnancy** | ☐ | No | ☐ | Unknown | ☐ | Yes |  |
| --- | --- | --- | --- | --- | --- | --- | --- |

If pregnancy yes:

| Date of Diagnosis: |  | |  | Expected date of delivery: | | |  |
| --- | --- | --- | --- | --- | --- | --- | --- |
|  |  |  |  |  |  |  |  |
| Gestational diabetes | ☐ | No | ☐ | Unknown | ☐ | Yes |  |
| Preclampsia/eclampsia | ☐ | No | ☐ | Unknown | ☐ | Yes |  |
| Fetal Macrosomy | ☐ | No | ☐ | Unknown | ☐ | Yes |  |
| Other complications |  |  |  |  |  |  |  |

| End of pregnancy | ☐ | miscarriage | ☐ | abortion | ☐ | birth | date: |
| --- | --- | --- | --- | --- | --- | --- | --- |
| If birth: | Birth weight: | | | kg | |  |  |
|  | Birth length: | | | cm | |  |  |
|  | APGAR 1 minute: | | |  | |  |  |
|  | APGAR minutes | | |  | |  |  |
|  | APGAR 10 minutes | | |  | |  |  |
|  | Neonatal complications: | | |  | |  |  |
|  | Congenital malformations: | | |  | |  |  |

If lactation: Begin of lactation:__________ End of lactation:_________________

**Organ systems - Haematology**

| ☐ | Not examined |  | ☐ | Normal | ☐ | Unchanged | ☐ | New or changed abnormalities |
| --- | --- | --- | --- | --- | --- | --- | --- | --- |

**If abnormal:**

| Anaemia | ☐ | No | ☐ | Unknown | ☐ | Yes | age at onset: |
| --- | --- | --- | --- | --- | --- | --- | --- |
| Coagulation abnormalities | ☐ | No | ☐ | Unknown | ☐ | Yes | age at onset: |
| Increased erythrocyte sedimentation rate | ☐ | No | ☐ | Unknown | ☐ | Yes | age at onset: |
| Hypergammaglobulinemia | ☐ | No | ☐ | Unknown | ☐ | Yes | age at onset: |
| Thrombocytosis | ☐ | No | ☐ | Unknown | ☐ | Yes | age at onset: |
| Thrombocytopenia | ☐ | No | ☐ | Unknown | ☐ | Yes | age at onset: |
| Tumors | ☐ | No | ☐ | Unknown | ☐ | Yes | type of tumor  age at onset: |
| Others |  |  |  |  |  |  | age at onset: |

**Organ systems - Immune System**

| ☐ | Not examined |  | ☐ | Normal | ☐ | Unchanged | ☐ | New or changed abnormalities |
| --- | --- | --- | --- | --- | --- | --- | --- | --- |

**If abnormal:**

| Celiac disease | ☐ | No | ☐ | Unknown | ☐ | Yes | age at onset: |
| --- | --- | --- | --- | --- | --- | --- | --- |
| Juvenile dermatomyositis | ☐ | No | ☐ | Unknown | ☐ | Yes | age at onset: |
| Autoimmune hepatitis | ☐ | No | ☐ | Unknown | ☐ | Yes | age at onset:  type: |
| Glomerulonephritis | ☐ | No | ☐ | Unknown | ☐ | Yes | age at onset:  type: |
| Tumors | ☐ | No | ☐ | Unknown | ☐ | Yes | type of tumor  age at onset: |
| Others |  |  |  |  |  |  | age at onset: |
| Antibodies |  |  |  |  |  |  |  |

**Psychosocial Health**

| ☐ | Not examined |  | ☐ | Normal | | | ☐ | | Unchanged | | ☐ | | New or changed abnormalities | | | |
| --- | --- | --- | --- | --- | --- | --- | --- | --- | --- | --- | --- | --- | --- | --- | --- | --- |
| Fatigue | | | | | ☐ | No | | ☐ | | Unknown | | ☐ | | Yes | age at onset: |  |
| Pain | | | | | ☐ | No | | ☐ | | Unknown | | ☐ | | Yes | age at onset: |  |
| Depression | | | | | ☐ | No | | ☐ | | Unknown | | ☐ | | Yes | age at onset: |  |
| Smoking | | | | | ☐ | No | | ☐ | | Unknown | | ☐ | | Yes | cigarettes/week: |  |
| Alcohol consumption | | | | | ☐ | No | | ☐ | | Unknown | | ☐ | | Yes | units/week: |  |

**Organ systems - Metabolic Systems**

| ☐ | Not examined |  | ☐ | Normal | ☐ | Unchanged | ☐ | New or changed abnormalities |
| --- | --- | --- | --- | --- | --- | --- | --- | --- |

**If abnormal:**

| Impaired glucose tolerance | ☐ | No | ☐ | Unknown | ☐ | Yes | age at onset: |
| --- | --- | --- | --- | --- | --- | --- | --- |
| Impaired fasting glucose | ☐ | No | ☐ | Unknown | ☐ | Yes | age at onset: |
| Diabetes mellitus Type 2 | ☐ | No | ☐ | Unknown | ☐ | Yes | age at onset: |
| Diabetic ketoacidosis | ☐ | No | ☐ | Unknown | ☐ | Yes | age at onset: |
| Hyooglycemia | ☐ | No | ☐ | Unknown | ☐ | Yes | age at onset: |
| Dyslipidemia | ☐ | No | ☐ | Unknown | ☐ | Yes | age at onset:  type: |
| Tumors | ☐ | No | ☐ | Unknown | ☐ | Yes | type of tumor:  age at onset: |
| Other diabetes subtypes | ☐ Mody 1 ☐ Mody 2 ☐ Mody 3 ☐ Mody 4 ☐ Mody 5 | | | | | | |
|  | ☐ Type 1 ☐ Gestational ☐ Others | | | | | | |
|  | age at onset of diabetes subtype: | | | | | | |

**Complications of DM**

| ☐ | Not examined |  | ☐ | None | | | ☐ | | Unchanged | | ☐ | | New or changed complications | | | |
| --- | --- | --- | --- | --- | --- | --- | --- | --- | --- | --- | --- | --- | --- | --- | --- | --- |
| Retinopathy | | | | | ☐ | No | | ☐ | | Unknown | | ☐ | | Yes | age at onset: |  |
| Nephropathy | | | | | ☐ | No | | ☐ | | Unknown | | ☐ | | Yes | age at onset: |  |
| Neuropathy | | | | | ☐ | No | | ☐ | | Unknown | | ☐ | | Yes | age at onset: |  |
| Macroangiopathy | | | | | ☐ | No | | ☐ | | Unknown | | ☐ | | Yes | age at onset: |  |
| Other complications | | | | |  | | | | | | | | | | age at onset: |  |

**Most recent clinical laboratory values**

| ☐ | Not examined | |  | ☐ | | Examined |  | | | | | |
| --- | --- | --- | --- | --- | --- | --- | --- | --- | --- | --- | --- | --- |
| Hemoglobin | | g/dl | | |  | Fasting plasma glucose | | mmol/l |  | Fasting serum triglycerides | mmol/l |  |
| WBC | | Giga/l | | |  | Fasting insulin | | mU/l |  | Total cholesterol | mmol/l |  |
| Platelets | | Giga/l | | |  | Fasting C peptide | | nmol/l |  | HDL cholesterol | mmol/l |  |
| Albumin | | g/l | | |  | HbA1c | | % |  | LDL cholesterol | mmol/l |  |
| Calcium | | mmol/l | | |  | Leptin | | ug/l |  | 25 OH vitamin D | ng/ml |  |
| Creatin kinase | | U/l | | |  | Adiponectin | | ug/ml |  | PTH (total) | pg/ml |  |
| ALT | | U/l | | |  | hsCRP | | mg/l |  | C3 complement | mg/dl |  |
| AST | | U/l | | |  | Serum creatinine | | umol/l |  | C3NeF | g/l |  |
| Gamma GT | | U/l | | |  | Estimated GFR | | ml/min/  1.73m^2^ |  | TSH | mU/l |  |
| Bilirubin | | U/l | | |  | Serum urea | | mmol/l |  | Urine protein | mg/l |  |

**Medications**

|  | ☐ | No medication | | ☐ | Unchanged | | | ☐ | New or changed medication | | | |  | |
| --- | --- | --- | --- | --- | --- | --- | --- | --- | --- | --- | --- | --- | --- | --- |
| Drug  (ATC code): | | |  | | |  | absolute daily  dose (unit): | | |  |  | starting date: | |  |

| Drug  (ATC code): |  |  | absolute daily  dose (unit): |  |  | starting date: |  |
| --- | --- | --- | --- | --- | --- | --- | --- |

| Drug  (ATC code): |  |  | absolute daily  dose (unit): |  |  | starting date: |  |
| --- | --- | --- | --- | --- | --- | --- | --- |
